# Analysis of Epigenetic Age Acceleration and Healthy Longevity Among Older US Women

**DOI:** 10.1001/jamanetworkopen.2022.23285

**Published:** 2022-07-27

**Authors:** Purva Jain, Alexandra M. Binder, Brian Chen, Humberto Parada, Linda C. Gallo, John Alcaraz, Steve Horvath, Parveen Bhatti, Eric A. Whitsel, Kristina Jordahl, Andrea A. Baccarelli, Lifang Hou, James D. Stewart, Yun Li, Jamie N. Justice, Andrea Z. LaCroix

**Affiliations:** 1 Herbert Wertheim School of Public Health and Human Longevity Science, University of California San Diego, La Jolla; 2 Cancer Epidemiology Program, University of Hawaii Cancer Center, Honolulu; 3 Department of Epidemiology, Fielding School of Public Health, University of California, Los Angeles; 4 Division of Epidemiology and Biostatistics, School of Public Health, San Diego State University; 5 Moores Cancer Center, University of California, San Diego, La Jolla; 6 Department of Human Genetics, David Geffen School of Medicine, University of California, Los Angeles; 7 Department of Biostatistics, School of Public Health, University of California, Los Angeles; 8 Cancer Control Research, BC Cancer, Vancouver, British Columbia, Canada; 9 Department of Epidemiology, Gillings School of Public Health, Chapel Hill, North Carolina; 10 Department of Medicine, School of Medicine, University of North Carolina at Chapel Hill; 11 Department of Epidemiology, School of Public Health, University of Washington, Seattle; 12 Department of Environmental Health Sciences, Mailman School of Public Health, Columbia University Irving Medical Center, New York, New York; 13 Institute for Public Health and Medicine, Northwestern University, Chicago, Illinois; 14 Department of Genetics, University of North Carolina at Chapel Hill; 15 Department of Biostatistics, University of North Carolina at Chapel Hill; 16 Department of Computer Science, University of North Carolina at Chapel Hill; 17 Sticht Center for Healthy Aging and Alzheimer’s Prevention, Section of Gerontology and Geriatric Medicine, Department of Internal Medicine, Wake Forest School of Medicine, Winston-Salem, North Carolina

## Abstract

**Importance:**

Accelerated biological aging is associated with decreased physical capability and cognitive functioning, which are associated with increased risk of morbidity and mortality.

**Objective:**

We investigated associations between epigenetic age acceleration (EAA), a biomarker associated with aging, and healthy longevity among older women.

**Design, Setting, and Participants:**

This cohort study was a secondary analysis of participants in the Women’s Health Initiative (WHI) who were eligible to survive to age 90 years by September 30, 2020. Participants were located in multiple centers. This study was restricted to women with genome-wide DNA methylation data, generated from baseline blood samples within 3 WHI ancillary studies. Median (IQR) follow-up times from baseline were 21.6 (19.6-22.9) years and 21.4 (19.8-22.7) years for women who survived to age 90 years with and without intact mobility, respectively, and 13.2 (8.8-16.7) for women who did not survive to age 90 years. Data were analyzed from December 2020 to July 2021.

**Exposures:**

EAA was estimated using 4 established “clocks”: Horvath pantissue, Hannum, Pheno, and Grim.

**Main Outcomes and Measures:**

Using multinomial logistic regression, odds ratios (ORs) and 95% CIs were estimated for 3 healthy longevity outcomes for each clock: survival to age 90 years with intact mobility, survival to age 90 years without intact mobility, and no survival to age 90 years.

**Results:**

Among 1813 women, there were 464 women (mean [SD] age at baseline, 71.6 [3.5] years) who survived to age 90 years with intact mobility and cognitive functioning, 420 women (mean [SD] age at baseline, 71.3 [3.2] years) who survived to age 90 years without intact mobility and cognitive functioning, and 929 women (mean [SD] age at baseline, 70.2 [3.4] years) who did not survive to age 90 years. Women who survived to age 90 years with intact mobility and cognitive function were healthier at baseline compared with women who survived without those outcomes or who did not survive to age 90 years (eg, 143 women [30.8%] vs 101 women [24.0%] and 202 women [21.7%] with 0 chronic conditions). The odds of surviving to age 90 years with intact mobility were lower for every 1 SD increase in EAA compared with those who did not survive to age 90 years as measured by AgeAccelHorvath (OR, 0.82; 95% CI, 0.69-0.96; *P* = .01), AgeAccelHannum (OR, 0.67; 95% CI, 0.56-0.80; *P* < .001), AgeAccelPheno (OR, 0.60; 95% CI, 0.51-0.72; *P* < .001), and AgeAccelGrim (OR, 0.68; 95% CI, 0.55-0.84; *P* < .001). ORs were similar for women who survived to age 90 years with intact mobility and cognitive function (eg, AgeAccelHorvath: OR per 1 SD increase in EAA, 0.83; 95% CI, 0.71-0.98; *P* = .03) compared with women who did not survive to age 90 years.

**Conclusions and Relevance:**

These findings suggest that EAA may be a valid biomarker associated with healthy longevity among older women and may be used for risk stratification and risk estimation of future functional and cognitive aging. Outcomes suggest that future studies may focus on the potential for public health interventions to counteract EAA and its association with poor health outcomes to lower disease burden while increasing longevity.

## Key Points

**Question:**

Is epigenetic age acceleration (accelerated biological aging) associated with healthy longevity among older women?

**Findings:**

This cohort study was a secondary analysis of 3 Women’s Health Initiative ancillary studies among 1813 women eligible to survive to age 90 years by end of study period. The study found that increased epigenetic age acceleration as measured by 4 epigenetic clocks was associated with lower odds of survival to age 90 years with intact mobility; results were similar when including intact cognitive functioning.

**Meaning:**

These findings suggest that epigenetic age acceleration may be a useful biomarker to estimate functional and cognitive aging among older women.

## Teaser

This cohort study investigates the association of epigenetic age acceleration with odds of surviving to age 90 with intact function among older US women.

## Introduction

The number of individuals aged 90 years and older is expected to quadruple, from 1.9 million in 2016 to 7.6 million in 2050 in the United States.^1^ Traditionally, those aged 85 years or older have been considered the oldest among older individuals. However, increases in life expectancy suggest that focus should turn to those who aged 90 years or older. Women make up a significantly larger proportion of long-lived individuals, outnumbering men 3 to 1 among those aged 90 years or older.^2^

Maintaining physical and mental capabilities is the foundation of well-being in older age.^3,4^ Biological aging encompasses changes in underlying hallmarks of aging, including epigenetics, which are associated with health trajectories and risk of morbidity and mortality.^5^ Individuals with healthy longevity are thought to have a biological age that is less than their chronological age. Moreover, among individuals of the same chronological age, there is considerable heterogeneity in physiologic functions and rate of biological aging.^6^

Epigenetic age is a biomarker of aging previously reported to be associated with age-related disease and all-cause mortality.^7,8,9,10^ It is a composite measure of DNA methylation (DNAm) levels across specific cytosine-guanine dinucleotide (CpG) sites that together form a single measure associated with chronological or phenotypic age. Epigenetic age acceleration (EAA), the residual variation in epigenetic age independent of chronological age, is 1 measure of whether individuals are aging faster or slower than their chronological age. EAA signifies individuals who, owing to a combination of endogenous and exogenous factors, are aging faster biologically compared with their chronological age, whereas inverse or slower age acceleration signifies the opposite. Prior studies suggest that slower age acceleration occurs among long-lived individuals.^11,12,13^ Older epigenetic age was also reported to be associated with lower levels of physical functioning^9,10^ and declines in global cognitive functioning among long-lived individuals.^14,15,16^

The aims of this study, therefore, were to investigate associations between EAA and healthy longevity. This was defined as survival to age 90 years with intact mobility and survival to age 90 years with intact mobility and cognitive functioning.

## Methods

This cohort study’s protocols were approved by the Fred Hutchinson Cancer Center Institutional Review Board, and all study participants provided informed consent in writing or by phone. Findings are reported in alignment with the Strengthening the Reporting of Observational Studies in Epidemiology (STROBE) reporting guideline.

### Study Population

The Women’s Health Initiative (WHI) began in 1993 with the goal of identifying strategies to prevent heart disease, osteoporosis, and breast and colorectal cancer among 161 808 postmenopausal women.^17,18^ This cohort study included participants from 3 WHI ancillary studies that had DNAm data available. The Bladder Cancer and Leukocyte Methylation ancillary study (study 1) included 468 individuals with bladder cancer and a control group of 468 matched individuals without cancer to identify methylation profiles associated with cancer risk.^19^ The Epigenetic Mechanisms of Particulate Matter–Mediated Cardiovascular Disease ancillary study (study 2) included a random sample of 2200 WHI clinical trial participants to understand the pathophysiological mechanisms associated with particulate matter–related cardiovascular disease in postmenopausal women.^20^ Lastly, the Integrative Genomics for Risk of Coronary Heart Disease and Related Phenotypes in the WHI Cohort ancillary study (study 3) included 1070 women with and 1070 women without coronary heart disease to integrate biomarkers into diagnostic and prognostic predictors of CHD and related phenotypes.^21^ DNAm was evaluated before diagnosis of incident bladder cancer and incident CHD.

Across the 3 ancillary studies, 2079 women survived to age 90 years as of September 30, 2020. Of these, 1819 women (87.5%) had information available on all physical and cognitive longevity components (eFigure 1 in the Supplement).

### Measures

#### Epigenetic Age

In each ancillary study, DNAm was measured using the Illumina Infinium 450K platform (Illumina). The minfi package version 3.15 for R statistical software version 4.12 (R Project for Statistical Computing) was used to read in all DNAm data files, check for failed samples, and implement normalization and quality control steps. Basic quality controls excluded probes targeting CpG sites on the Y chromosome, with detection *P* values > .01 in more than 1% of samples, with a bead count of less than 3 in more than 10% of samples, and that measure non-CpG methylation. Normalization was completed using β-mixture quantile normalization and implemented using the beta mixture quantile procedure in the wateRmelon package version 3.14 in R statistical software version 1.4.1106.^22^ Epigenetic age was estimated using 4 established clocks, including the Horvath pantissue, Hannum, Pheno, and Grim clocks, as summarized in eTable 1 in the Supplement.

#### Survival Outcomes

The first outcome focused on mobility as follows: (1) survival to age 90 years with intact mobility or (2) survival to age 90 years with impairment in mobility vs (3) death before age 90 years. The second outcome additionally incorporated cognitive function as follows: (1) survival to age 90 years with intact mobility and cognition or (2) survival to age 90 years with impairment in mobility, cognition, or both vs (3) death before age 90 years (Table 1). At the time of this analysis, the median age of death in WHI participants was near 90 years, and thus age 90 years was considered the threshold age for defining healthy longevity. Survival to age 90 years was calculated from day of enrollment in WHI through September 30, 2020, and death before age 90 years was used as the reference group for all analyses. WHI ascertained death using annual mailed outcome questionnaires and systematic searches of the National Death Index, hospital records, obituaries, and proxy queries.^23^ Intact mobility was defined using 2 questions from the RAND-36 Physical Function questionnaire^24^ as having no or little self-reported limitations for walking 1 block and climbing 1 flight of stairs at the closest measure prior to age 90 years. The questionnaire was administered at baseline, at 1-year and 3-year follow-up assessments, and then annually after 2005. Intact cognitive functioning was ascertained through annual surveillance of self-reported moderate or severe memory problems or physician-diagnosed dementia or Alzheimer disease prior to age 90 years. If either of these conditions was reported, women were classified as having cognitive impairment.

**Table 1.** Healthy Longevity Outcome Components and Comparison Groups

Outcome	Healthy longevity outcome group		
	Group 1	Group 2	Group 3 (reference)
Outcome 1: longevity + physical health	Survival to age 90 y with intact mobility^a^^,^^b^	Survival to age 90 y and loss of intact mobility	No survival to age 90 y
Outcome 2: longevity + physical and cognitive health	Survival to age 90 y with intact mobility and cognitive function^c^	Survival to age 90 y and loss of intact mobility, cognitive function, or both	No survival to age 90 y
Sensitivity analysis: longevity + physical and cognitive health (WHIMS)	Survival to age 90 y with intact mobility and cognitive function^d^	Survival to age 90 y and loss of intact mobility, cognitive function, or both	No survival to age 90 y

Abbreviation: WHIMS, Women’s Health Initiative Memory Study.

^a^ Survival to age 90 years is defined occurring from Women’s Health Initiative baseline to end of follow-up.

^b^ Intact mobility is defined as no report of “Yes, limited a lot” or “Yes, limited a little” to walk 1 block or climb 1 flight of stairs on annual questionnaires from Women’s Health Initiative baseline to age 90 years.

^c^ Intact cognitive function is defined as no report of “Moderate or severe memory problems” or “Dementia or Alzheimer” on annual questionnaire from Women’s Health Initiative baseline to age 90 years.

^d^ Intact cognitive function is defined as no report of adjudicated diagnosis of “Probable dementia” from baseline to age 90 years in Women’s Health Initiative Memory Study.

Among women enrolled in the WHI Extension Study 1 (2005-2010) with at least 1 outcomes form collected after enrollment, this classification of Alzheimer disease vs Medicare claims had a sensitivity of 40% and specificity of 95%. The Women’s Health Initiative Memory Study (WHIMS) is a subcohort of WHI women aged 65 years and older who participated in the Hormone Therapy Trial.^25^ WHIMS investigated incidence of all-cause dementia using cognitive functioning screening and neurologic and neuropsychological evaluations followed by surveillance for changes in cognitive functioning and use of a consensus panel to define probable dementia. Self-reported dementia or Alzheimer disease compared with WHIMS classification had a sensitivity of 41% and specificity of 89%.

#### Covariates

Covariates were measured at baseline, with the exception of age, which was measured at blood draw. We selected covariates owing to their associations in the literature with EAA and healthy longevity. Covariates included age, estimated blood cell composition using the Houseman method^26^ (CD8+ T cells, CD4 T cells, natural killer cells, B lymphocyte cells, monocytes, and granulocytes), race and ethnicity (Black or African American, Hispanic or Latino, White and not of Hispanic origin, and unknown [ie, not one of the previous categories]), education (high school or general education development or less, some college, and college graduate or more), walking frequency (rarely or never, 1-3 times/mo, 1 time/wk, 2-3 times/wk, 4-6 times/wk, and ≥7 times/wk), body mass index (BMI; calculated as weight in kilograms divided by height in meters squared) category (underweight [<18.5], reference range [18.5-24.9], overweight [25.0-29.9], and obese [≥30]), alcohol consumption (nondrinker, past drinker, <1 drink/mo, <1 drink/wk, 1 to <7 drinks/wk, and ≥7 drinks/wk), smoking history (never smoker and <5, 5-20, and ≥20 pack-years), number of chronic conditions at baseline (0, 1-2, and ≥3, including cancer, stroke, Alzheimer disease, cardiovascular disease, diabetes, history of frequent falls [≥2/y], broken hip, emphysema, arthritis, depression, urinary incontinency, and visual or auditory sensory impairment), and physical function score (RAND-36 10-item physical function subscale^24^; range, 0-100; higher score reflects higher function). Chronic conditions were chosen based on the association of these conditions with a high degree of changes in lifespan and health span of older women.^27,28,29^ Race and ethnicity were self-reported by participants from categories listed on a questionnaire (Black or African-American, Hispanic or Latino, and White [not of Hispanic origin]). Race and ethnicity were assessed in the larger WHI to allow investigation of health disparities and used in this study to control for potential confounding.

### Statistical Analysis

Analyses were conducted using R statistical software version 1.4.1106 (R Project for Statistical Computing). Baseline characteristics were reported by healthy longevity category, and differences by category were tested using Pearson χ^2^ tests for categorical variables and *F* tests for continuous variables. Correlations between chronological age and each DNAm age measure were evaluated using Pearson correlation coefficient. Fully adjusted multinomial logistic regression models with a random intercept for ancillary study were used to estimate odds ratios (ORs) and 95% CIs for associations between SD increases in each EAA measure and healthy longevity. The adjusted model included all covariates as described previously and inverse probability weights to account for case-control sampling of study 1 and study 3 and for oversampling of racial and ethnic minority groups in study 2. Weights were the inverse of the selection probability for each individual, and contributions of oversampled cases were accordingly downweighted. The sample was reweighted so that the sum of the weights was similar to the original sample size. We used 2-sided statistical tests with α = .05. Data were analyzed from December 2020 to July 2021.

We examined the association of EAA as estimated by each clock with healthy longevity outcomes as follows: (1) women who survived to age 90 years with intact mobility and women who survived to age 90 years with intact mobility and cognitive function compared with women who did not survive to age 90 years and (2) women who survived to age 90 years without intact mobility, cognitive functioning, or both compared with women who did not survive to age 90 (Table 1).

Subgroup analyses by baseline age (median split = 70.5 years) were completed in fully adjusted and weighted, pooled multinomial logistic regression models and tested using interaction terms with Wald test at an α of .05. Additionally, results from fully adjusted and weighted models were stratified by ancillary study (eTable 3 in the Supplement).

Given prior evidence of a positive correlation between concurrently measured physical activity and EAA,^14,30^ our primary analysis adjusted for these characteristics at baseline to investigate if EAA was independently associated with survival to age 90 years with intact mobility. Because the mobility outcome has been found to be associated with physical function, sensitivity analyses excluding baseline walking frequency and physical function score from models were conducted. In addition, sensitivity analyses were conducted by replacing the WHI self-reported measure of cognitive impairment with an adjudicated diagnosis of probable dementia from WHIMS. The analysis was limited to women who participated in WHI and WHIMS.

## Results

Among 1813 women, there were 464 women (mean [SD] age at baseline, 71.6 [3.5] years) who experienced healthy longevity (ie, survived to age 90 years with intact mobility and cognitive functioning); 420 women (mean [SD] age at baseline, 71.3 [3.2] years) who survived to age 90 years without intact mobility, cognitive functioning, or both; and 929 women (mean [SD] age at baseline, 70.2 [3.4] years) who did not survive to age 90 years. Women who experienced healthy longevity, compared with women who survived to age 90 years without intact function and women who did not survive to age 90 years, were more likely to be White and not of Hispanic Origin. There were 66 Black individuals (14.3%), 27 Hispanic or Latino individuals (5.9), and 348 (75.7) White individuals not of Hispanic origin among women with healthy longevity; 73 Black individuals (17.4%), 36 Hispanic or Latino individuals (8.6%), and 305 White individuals not of Hispanic origin (72.8%) among women who survived to age 90 years without intact function; and 179 Black individuals (19.4%), 78 Hispanic or Latino individuals (8.5%), and 637 White individuals not of Hispanic origin (69.1%) among women who did not survive to age 90 years. Those with healthy longevity were also more likely to have none of the major chronic conditions examined (143 women [30.8%] vs 101 women [24.0%] and 202 women [21.7%]) compared with women who survived to age 90 years without intact function and those who did not survive to age 90 years. Women surviving with healthy longevity were also more likely to be college graduates, have no history of smoking, walk 2 to 3 times per week or 4 to 6 times per week, have a BMI in the reference or overweight range, and have more than 1 but fewer than 7 alcoholic drinks per week; they additionally had a higher mean (SD) physical functioning score (Table 2). Median (IQR) follow-up times from WHI enrollment to age 90 years or time of death were 21.6 (19.6-22.9) years for those who survived to age 90 years with intact mobility, 21.4 (19.8-22.7) years for those who survived to age 90 years without intact function, and 13.2 (8.8-16.7) years for those who did not survive to age 90 years.

**Table 2.** Baseline Characteristics by Outcome Level

Characteristic	Women, No. (%) (N = 1813)			*P* value
	Survived to age 90 y		Did not survive to age 90 y (n = 929)	
	With intact mobility and cognitive functioning (n = 464)	Without intact mobility and cognitive functioning (n = 420)		
Age at baseline, mean (SD), y	71.6 (3.5)	71.3 (3.2)	70.2 (3.4)	<.001
Race and ethnicity				
Black or African American	66 (14.3)	73 (17.4)	179 (19.4)	.01
Hispanic or Latino	27 (5.9)	36 (8.6)	78 (8.5)	
White and not of Hispanic origin	348 (75.7)	305 (72.8)	637 (69.1)	
Unknown	19 (4.1)	5 (1.2)	28 (3.0)	
Education				
≤High school or GED	103 (22.3)	114 (27.3)	281 (30.5)	.003
Some college	181 (39.2)	177 (42.3)	369 (40.0)	
≥College grad	178 (38.5)	127 (30.4)	272 (29.5)	
Walking frequency				
Rarely or never	56 (12.1)	88 (21.2)	204 (22.2)	<.001
1-3 Times/mo	66 (14.2)	51 (12.3)	144 (15.7)	
1 Time/week	50 (10.8)	34 (8.2)	109 (11.9)	
2-3 Times/week	144 (31.0)	118 (28.4)	231 (25.2)	
4-6 Times/week	111 (23.9)	90 (21.7)	163 (17.8)	
≥7 Times/week	37 (8.0)	34 (8.2)	67 (7.3)	
BMI category				
Underweight (<18.5)	6 (1.3)	3 (0.7)	9 (1.0)	<.001
Normal (18.5-24.9)	167 (36.2)	101 (24.2)	251 (27.2)	
Overweight (25.0-29.9)	189 (41.0)	150 (35.9)	296 (32.0)	
Obese (≥30)	99 (21.5)	164 (39.2)	368 (39.8)	
Alcohol consumption				
Nondrinker	60 (13.1)	63 (15.1)	121 (13.2)	<.001
Past drinker	70 (15.3)	95 (22.7)	224 (24.5)	
<1 Drink/mo	51 (11.1)	56 (13.4)	134 (14.6)	
<1 Drink/wk	99 (21.6)	93 (22.2)	167 (18.3)	
1 to <7 Drinks/wk	120 (26.1)	71 (17.0)	173 (18.9)	
≥7 Drinks/wk	59 (12.9)	40 (9.6)	96 (10.5)	
Smoking, pack-years				
Never smoker	277 (62.0)	248 (60.6)	425 (47.6)	<.001
<5	51 (11.4)	59 (14.4)	96 (10.8)	
5 to <20	64 (14.3)	41 (10.0)	114 (12.8)	
≥20	55 (12.3)	61 (14.9)	258 (28.9)	
Chronic conditions, No.^a^				
0	143 (30.8)	101 (24.0)	202 (21.7)	<.001
1-2	291 (62.7)	271 (64.5)	615 (66.2)	
≥3	30 (6.5)	48 (11.4)	112 (12.1)	
Physical function score, mean (SD)	82.4 (20.2)	72.8 (22.7)	69.5 (24.6)	<.001
EAA measure, mean (SD), y^b^				
AgeAccelHorvath	−0.6 (5.3)	0.02 (5.4)	0.09 (5.3)	.05
AgeAccelHannum^b^	−1.2 (4.9)	0.1 (5.0)	0.4 (5.2)	<.001
AgeAccelGrim^b^	−1.5 (6.8)	0.5 (6.8)	1.1 (7.0)	<.001
AgeAccelPheno^b^	−1.3 (3.4)	−0.6 (3.5)	0.8 (4.2)	<.001

Abbreviations: BMI, body mass index (calculated as weight in kilograms divided by height in meters squared); EAA, epigenetic age acceleration; GED, general educational development.

^a^ Conditions include cardiovascular disease, cancer, cognitive impairment, depression, osteoarthritis, history of falls, chronic obstructive pulmonary disease, hypertension, diabetes, hip fracture, and cerebrovascular disease.

^b^ EAA measures are the residual between chronological age and epigenetic age as measured by epigenetic clock.

### Survival With Intact Function

**Figure 1.**
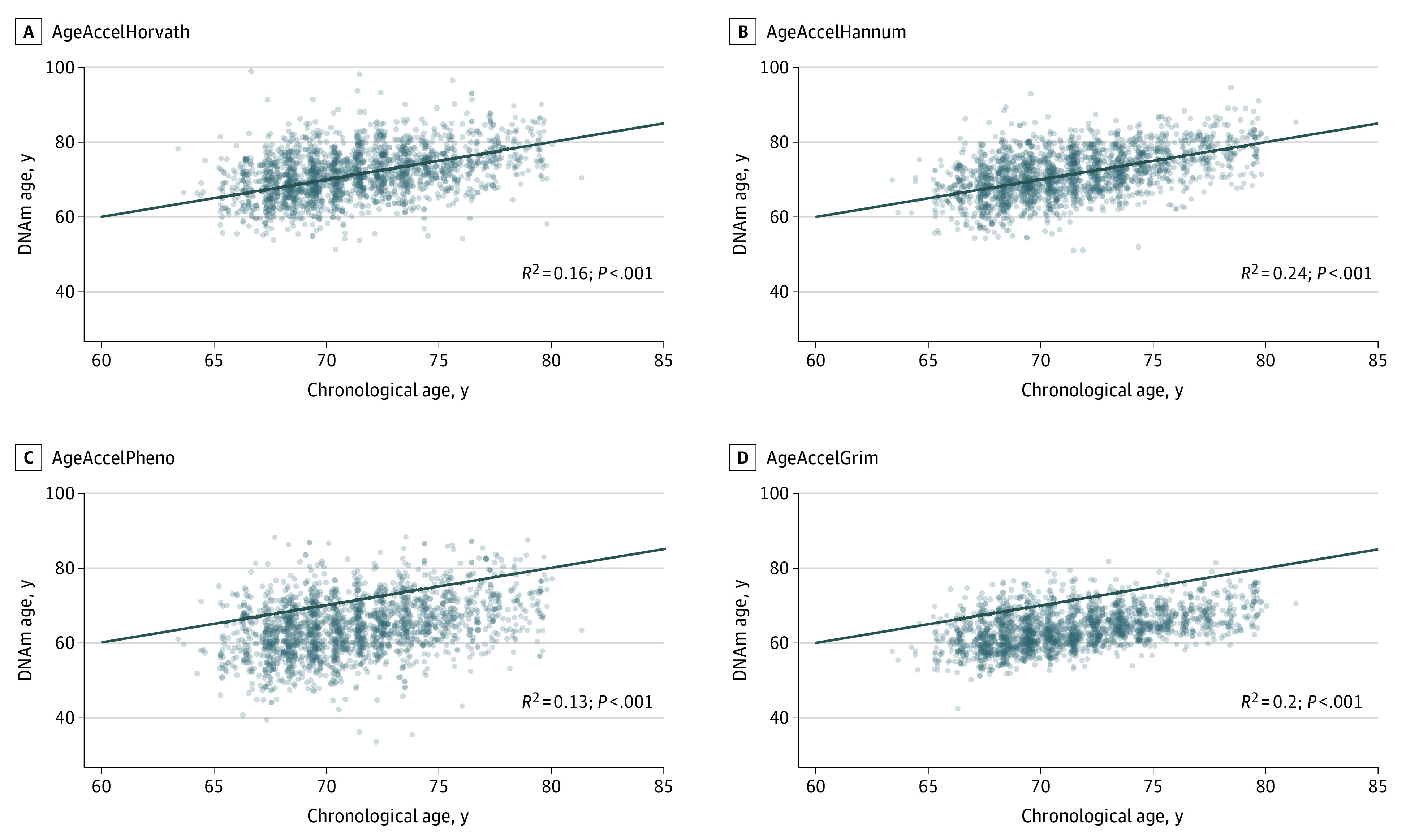
Correlation of Chronological Age and DNA Methylation (DNAm) Age Measures

There were 493 women and 391 women who survived to age 90 years with and without intact mobility, respectively, and 929 women who did not survive to age 90. There were 29 women who were reclassified from the healthy longevity group once intact cognitive function was included in the outcome definition. Results from multinomial logistic regression models examining associations between EAA and healthy longevity outcomes are reported in Table 3. Four epigenetic age measures were correlated with chronological age (Figure 1) and with each other (eFigure 2 in the Supplement). The odds of surviving to age 90 years with intact mobility were lower for every 1 SD increase in EAA compared with those who did not survive to age 90 years as measured by AgeAccelHorvath (OR, 0.82; 95% CI, 0.69-0.96; *P* = .01), AgeAccelHannum (OR, 0.67; 95% CI, 0.56-0.80; *P* < .001), AgeAccelPheno (OR, 0.60; 95% CI, 0.51-0.72; *P* < .001), and AgeAccelGrim (OR, 0.68; 95% CI, 0.55-0.84; *P* < .001). Outcomes were similar when for women who survived to age 90 years with intact mobility and cognitive function for every 1 SD increase in EAA vs women who did not survive to age 90 years as measured by AgeAccelHorvath (OR, 0.83; 95% CI, 0.71-0.98; *P* = .03), AgeAccelHannum (OR, 0.68; 95% CI, 0.57-0.82; *P* < .001), AgeAccelPheno (OR, 0.60; 95% CI, 0.50-0.72; *P* < .001), and AgeAccelGrim (OR, 0.73; 95% CI, 0.59-0.90; *P* = .003).

**Table 3.** Association of EAA and Healthy Longevity Outcomes

EAA measure^b^	Women who survived to age 90 y (N = 1813)			
	With healthy longevity^a^		Without healthy longevity^a^	
	OR (95% CI)^c^	*P* value	OR (95% CI)^c^	*P* value
**Mobility^d^**				
AgeAccelHorvath	0.82 (0.69-0.96)	.01	0.96 (0.81-1.15)	.68
AgeAccelHannum	0.67 (0.56-0.80)	<.001	0.96 (0.81-1.15)	.68
AgeAccelPheno	0.60 (0.51-0.72)	<.001	0.75 (0.63-0.90)	.002
AgeAccelGrim	0.68 (0.55-0.84)	<.001	0.82 (0.65-1.02)	.07
**Mobility and cognitive functioning^e^**				
AgeAccelHorvath	0.83 (0.71-0.98)	.03	0.93 (0.78-1.10)	.38
AgeAccelHannum	0.68 (0.57-0.82)	<.001	0.91 (0.77-1.09)	.31
AgeAccelPheno	0.60 (0.50-0.72)	<.001	0.74 (0.62-0.88)	.001
AgeAccelGrim	0.73 (0.59-0.90)	.003	0.75 (0.60-0.92)	.007

Abbreviations: EAA, epigenetic age acceleration; OR, odds ratio.

^a^ The reference group for all comparisons was 929 women who did not survive to age 90 years.

^b^ Models were adjusted for the following baseline covariates: blood cell composition (CD8 T, CD4 T, natural killer, B cell, monocyte, and granulocyte), age, race and ethnicity, education, walking frequency, body mass index (calculated as weight in kilograms divided by height in meters squared), alcohol consumption, pack-years smoking, number of chronic conditions (including cancer, stroke, Alzheimer disease, cardiovascular disease, diabetes, history of frequent falls [≥2/y], broken hip, emphysema, arthritis, depression, urinary incontinency, and visual or auditory sensory impairment), and RAND physical functioning score.

^c^ Results are presented for 1 SD increase in DNA methylation age measure: AgeAccelHorvath (SD = 6.4 years), AgeAccelHannum (SD = 6.2 years), AgeAccelPheno (SD = 7.6 years), and AgeAccelGrim (SD = 5.1 years).

^d^ There were 493 women with intact mobility and 391 women without intact mobility.

^e^ There were 464 women with intact mobility and cognitive functioning and 420 women without intact mobility, cognitive functioning, or both.

### Survival Without Intact Function

**Figure 2.**
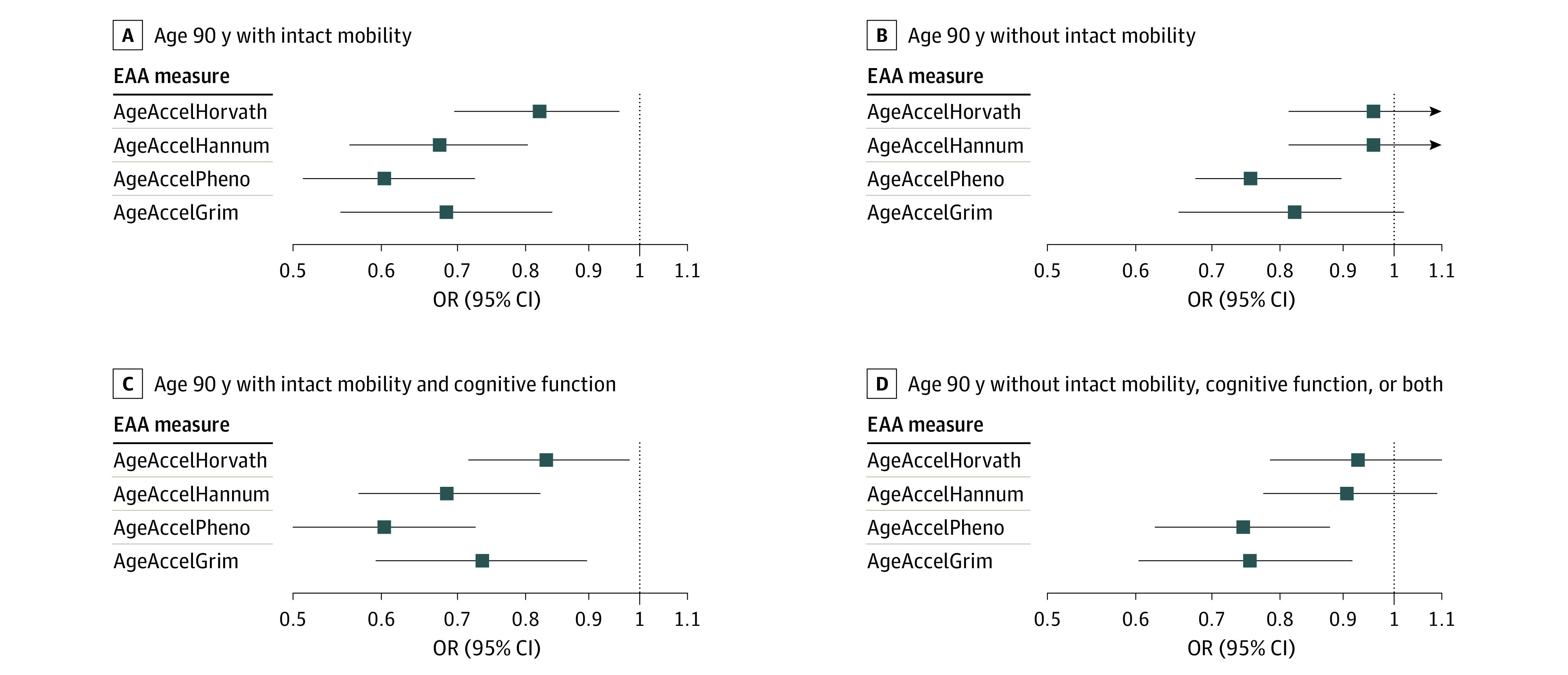
Forest Plots of the Association of EAA Measures and Healthy Longevity EAA indicates epigenetic age acceleration; OR, odds ratio.

The odds of surviving to age 90 years without intact mobility were lower for every 1 SD increase in EAA compared with women who did not survive to age 90 years as measured by AgeAccelPheno (OR, 0.75; 95% CI, 0.63-0.90; *P* = .002) and AgeAccelGrim (OR, 0.82; 95% CI, 0.65-1.02; *P* = .07) (Table 3 and Figure 2). These associations were consistent for the odds of surviving to age 90 years without intact mobility, cognitive functioning, or both for every 1 SD increase in EAA as measured by AgeAccelPheno (OR, 0.74; 95% CI, 0.62-0.88; *P* < .001) and AgeAccelGrim (OR, 0.75; 95% CI, 0.60-0.92; *P* = .007) (Table 3 and Figure 2).

Most outcomes remained significant after adjustment for multiple testing using a Bonferroni-adjusted *P* value threshold of .006. Primary results were similar when the fully adjusted model omitted walking frequency and physical function score. Additionally, results were similar when analyses were restricted to the subgroup of women who participated in WHIMS, in which the outcome of intact mobility and cognitive functioning was defined using an adjudicated measure of probable dementia or mild cognitive impairment (eTable 2 in the Supplement). Differences in outcomes were a higher OR in the association of AgeAccelPheno with odds of experiencing healthy longevity and no association for AgeAccelHorvath (eTable 2 in the Supplement). In secondary analyses, we investigated interactions of EAA measures with baseline age, which did not modify associations between EAA and healthy longevity (eTable 3 in the Supplement).

## Discussion

To our knowledge, this cohort study is the first study examining the association between EAA and healthy longevity among older women. In this racially and ethnically diverse cohort of older women, increased EAA as measured by AgeAccelHorvath, AgeAccelHannum, AgeAccelPheno, and AgeAccelGrim clocks was associated with lower odds of survival to age 90 years with intact mobility. Results were similar when including intact cognitive functioning, although only 29 women were reclassified from the healthy longevity group to surviving to age 90 years without intact mobility and cognitive functioning. Additionally, results remained similar when analyses were limited to the WHIMS population, in which an adjudicated measure for probable dementia and mild cognitive impairment was used.

Few studies have examined epigenetic aging in association with healthy longevity. Among 48 long-lived Nicoyans from the eponymously named peninsula of Costa Rica (mean age, 83 years) and 47 non-Nicoyans (mean age, 85 years), there were no statistically significant between-group differences observed for AgeAccelHorvath or AgeAccelHannum.^12^ The small sample size provided limited power to detect more modest differences, and several known differences between groups (eg, in education, health insurance, and adiposity) were not evaluated as potential confounders in the analysis.

Additional studies have investigated associations between EAA and physical and cognitive functioning among older adults, although these studies did not include long-lived individuals. One study included 791 members of the Lothian Birth Cohort 1936, a group of 1091 community-dwelling adults with a mean age of 70 years. The authors reported that a 1-year increase in extrinsic EAA was associated with a 6% increase in risk of being physically frail (ie, having ≥3 of the following characteristics: weakness, self-reported exhaustion, slow gait speed, unintentional weight loss, and low physical activity).^31^ When converted to a 6-year increase, the corresponding 42% increase in risk was within the range of our estimates (5.1-year to 7.6-year increases in epigenetic age). These findings were similar to those of a study^32^ that investigated this association among 1820 men and women aged 50 to 75 years. Another cross-sectional^14^ study, among 1091 individuals in the Lothian Birth Cohort, found an association between EAA and grip strength and fluid cognitive ability. Levine et al^15^ conducted a study of EAA and Alzheimer disease–related cognitive decline and associated neuropathological markers using 700 dorsolateral prefrontal cortex samples from non-Hispanic White individuals (mean age at enrollment, 81.4 years; mean age at death, 88.1 years) in the Religious Order Study and Rush Memory Aging Project and found an association between EAA of the dorsal prefrontal cortex and longitudinal decline in global cognitive functioning, episodic memory, and working memory among individuals with Alzheimer disease but not among those without Alzheimer disease.

Epigenetic clocks are measures of biological aging that were previously found to be associated with mortality, physical functioning, and cognitive status in addition to other markers of health.^7,8,9,10^ These clocks measure DNAm of cytosines at CpG nucleotides, which is 1 of the key epigenetic mechanisms involved in gene expression and splicing.^33^ Clocks differed in training methods, including age range, statistical methodology, sample characteristics, and technical factors used. First-generation clocks were trained to estimate chronological age and second-generation clocks to estimate multisystem phenotypic age and time to death.^33^ Training of AgeAccelPheno and AgeAccelGrim clocks to estimate the latter outcome most likely led to associations with larger ORs using newer clocks. There is a low overlap in CpGs and associated genes that are included in each clock, suggesting that aging has complex and varied involvement of different biological processes, such as transcription, epigenomic instability, telomere biology, and cellular differentiation and senescence.^34^ Associations in this study may be capturing these underlying biological processes and the influence of environmental factors as captured by epigenetic clocks.^35^

### Strengths and Limitations

This study has several limitations. It included only women, and replication in cohorts that include men and women, diverse racial and ethnic groups, and individuals from varied regions of the world may be important. Although it was of great interest to investigate the association between EAA and survival to age 90 years with intact cognitive function independently, this study population did not have sufficient numbers of women who experienced loss of cognitive function (without loss of mobility) to do so. This study benefitted from a large, racially and ethnically diverse sample of women who were followed up to at least age 90 years with detailed longitudinal data on a host of lifestyle and health history factors. Women were followed up for a mean of approximately 20 years with low rates of loss to follow-up. We used several chronological and phenotypic clocks to measure EAA. While inclusion of participants from ancillary studies using nested case-control designs could bias effect estimates, we used inverse probability selection weights to account for sampling structure to address potential biases related to sampling.^36^ This study is generalizable to the WHI women owing to the use of IPW weights and thus may be generalizable to a large range of women in the United States.

## Conclusions

This cohort study’s findings suggest that EAA may be a valid biomarker associated with healthy longevity among older women. Our results suggest that EAA may be used for risk stratification and risk estimation for future survival with intact mobility and cognitive functioning within populations. Future studies could usefully focus on the potential for public health interventions to reduce EAA and associated disease burden while increasing longevity.

## Notes

Supplement. **eTable 1.** Overview of Epigenetic Clocks Used **eTable 2.** Association of Epigenetic Age Acceleration and Healthy Longevity Using Women’s Health Initiative Memory Study Measure for Cognitive Impairment **eTable 3.** Interaction of Epigenetic Age Acceleration Measure With Baseline Age and Length of Follow-up in Primary Analysis **eFigure 1.** Study Flow Diagram **eFigure 2.** Correlation of DNA Methylation Measures **eFigure 3.** Forest Plots of Association of Epigenetic Age Acceleration Measures and Healthy Longevity by Ancillary Study
