# Supplementary material for: Analysis of Epigenetic Age Acceleration and Healthy Longevity Among Older US Women
Source: JAMA Netw Open. 2022 Jul 27;5(7):e2223285. doi: 10.1001/jamanetworkopen.2022.23285 (PMC9331104; doi:10.1001/jamanetworkopen.2022.23285)
Supplement: Supplement. — eTable 1. Overview of Epigenetic Clocks Used eTable 2. Association of Epigenetic Age Acceleration and Healthy Longevity Using Women’s Health Initiative Memory Study Measure for Cognitive Impairment eTable 3. Interaction of Epigenetic Age Acceleration Measure With Baseline Age and Length of Follow-up in Primary Analysis eFigure 1. Study Flow Diagram eFigure 2. Correlation of DNA Methylation Measures eFigure 3. Forest Plots of Association of Epigenetic Age Acceleration Measures and Healthy Longevity by Ancillary Study [file jamanetwopen-e2223285-s001.pdf]

Supplemental Online Content

Jain P, Binder AM, Chen B, et al. Analysis of epigenetic age acceleration and healthy longevity among older US women. *JAMA Netw Open*. 2022;5(7):e2223285. doi:10.1001/jamanetworkopen.2022.23285

eTable 1. Overview of Epigenetic Clocks Used
eTable 2. Association of Epigenetic Age Acceleration and Healthy Longevity Using Women's Health Initiative Memory Study Measure for Cognitive Impairment
eTable 3. Interaction of Epigenetic Age Acceleration Measure With Baseline Age and Length of Follow-up in Primary Analysis
eFigure 1. Study Flow Diagram
eFigure 2. Correlation of DNA Methylation Measures
eFigure 3. Forest Plots of Association of Epigenetic Age Acceleration Measures and Healthy Longevity by Ancillary Study

This supplemental material has been provided by the authors to give readers additional information about their work.

© 2022 Jain P et al. *JAMA Network Open.*

eTable 1. Overview of Epigenetic Clocks Used

Clock[a]	CpGs	Genes	Age	N	Tissue	Reported Associations
Horvath[7]	353	344	0-101	8000	Various cell & tissues	Chronological age, all-cause mortality, cancer, age-related disease and several neurodegenerative phenotypes
Hannum[8]	71	94	19-101	656	Blood	Chronological age, all-cause mortality
PhenoAge[9]	513	505	>20	9926	Blood	All-cause and cause-specific mortality, survival, count of comorbidities, physical functioning, smoking status and telomere length
GrimAge[10]	1030	NA	NA (mean=66)	1731	Blood	Morbidity and mortality, survival, cognitive decline, clinical biomarkers, lifestyle factors, blood cell composition and telomere length

[a]Table adapted from Bergsma & Rogaeva[34]

© 2022 Jain P et al. *JAMA Network Open.*

eTable 2. Association of Epigenetic Age Acceleration and Healthy Longevity Using Women's Health Initiative Memory Study Measure for Cognitive Impairment

	**90 with Intact Mobility & Cognitive Functioning (n=146)[b,c]**		**90 without Intact Mobility and/or Cognitive Functioning (n=195)[b,c]**	
EAA Measure[a]	OR (95% CI)[d]	p	OR (95% CI)[d]	p
**AgeAccelHorvath**	0.94 (0.63-1.37)	0.695	1.29 (0.88-1.74)	0.17
**AgeAccelHannum**	0.60 (0.37-0.94)	0.023	0.94 (0.64-1.44)	0.84
**AgeAccelPheno**	0.35 (0.15-0.73)	0.008	0.68 (0.35-1.25)	0.21
**AgeAccelGrim**	0.69 (0.52-0.90)	0.011	0.86 (0.69-1.11)	0.22

aAll models were adjusted for the following baseline covariates: blood cell composition (CD8T, CD4T, NK, Bcell, Mono, Gran), age, race/ethnicity, education, walking frequency, body mass index, alcohol consumption, pack-years smoking, number of chronic conditions (including cancer, stroke, Alzheimer's, cardiovascular disease, diabetes, history of frequent falls [2+/yr], broken hip, emphysema, arthritis, depression, urinary incontinency and visual/auditory sensory impairment) and RAND physical functioning score.

[b]This sensitivity analysis replaced the annual self-reported Women's Health Initiative measure of moderate or severe memory problems or Alzheimer's or dementia with the adjudicated Women's Health Initiative Memory Study measure of probable dementia.

[c]The reference group for all comparisons is women who did not survive to age 90 (n=325).

[d]Results are presented for one standard deviation increase in DNAmAge measure: AgeAccelHorvath (sd=6.4), AgeAccelHannum (sd=6.2), AgeAccelPheno (sd=7.6) and AgeAccelGrim (sd=5.1).

© 2022 Jain P et al. *JAMA Network Open.*

eTable 3. Interaction of Epigenetic Age Acceleration Measure With Baseline Age and Length of Follow-up in Primary Analysis

Follow-up: >70.6 years versus <=70.5 years

**Ref=Lower than median age at baseline (median=70.6 years)**	**90 with Intact Mobility & Cognitive Functioning (n=483)[b,c]**		**90 without Intact Mobility and/or Cognitive Functioning (n=403)[b,c]**	
EAA Measure[a]	OR (95% CI)[d]	p-int	OR (95% CI)[d]	p-int
**AgeAccelHorvath*median age**	1.14 (0.83-1.57)	0.376	1.29 (0.94-1.78)	0.13
**AgeAccelHannum*median age**	1.20 (0.65-1.13)	0.303	1.13 (0.83-1.54)	0.49
**AgeAccelPheno*median age**	0.93 (0.68-1.26)	0.654	1.16 (0.86-1.58)	0.37
**AgeAccelGrim*median age**	1.11 (0.77-1.50)	0.601	1.01 (0.74-1.43)	0.91

[a]All models were adjusted for the following baseline covariates: blood cell composition (CD8T, CD4T, NK, Bcell, Mono, Gran), age, race/ethnicity, education, walking frequency, BMI, alcohol consumption, pack-years smoking, number of chronic conditions (including cancer, stroke, Alzheimer's, cardiovascular disease, diabetes, history of frequent falls [2+/yr], broken hip, emphysema, arthritis, depression, urinary incontinency and visual/auditory sensory impairment) and RAND physical functioning score.

[b]The reference group for all comparisons is women who did not survive to age 90 (n=929).

[c]There were 1033 women with baseline median age <=70.5 years (reference) and 1045 women with baseline age >70.5 years.

[d]Results are presented for one standard deviation increase in DNAmAge measure: AgeAccelHorvath (sd=6.4), AgeAccelHannum (sd=6.2), AgeAccelPheno (sd=7.6) and AgeAccelGrim (sd=5.1).

© 2022 Jain P et al. *JAMA Network Open.*

eFigure 1. Study Flow Diagram

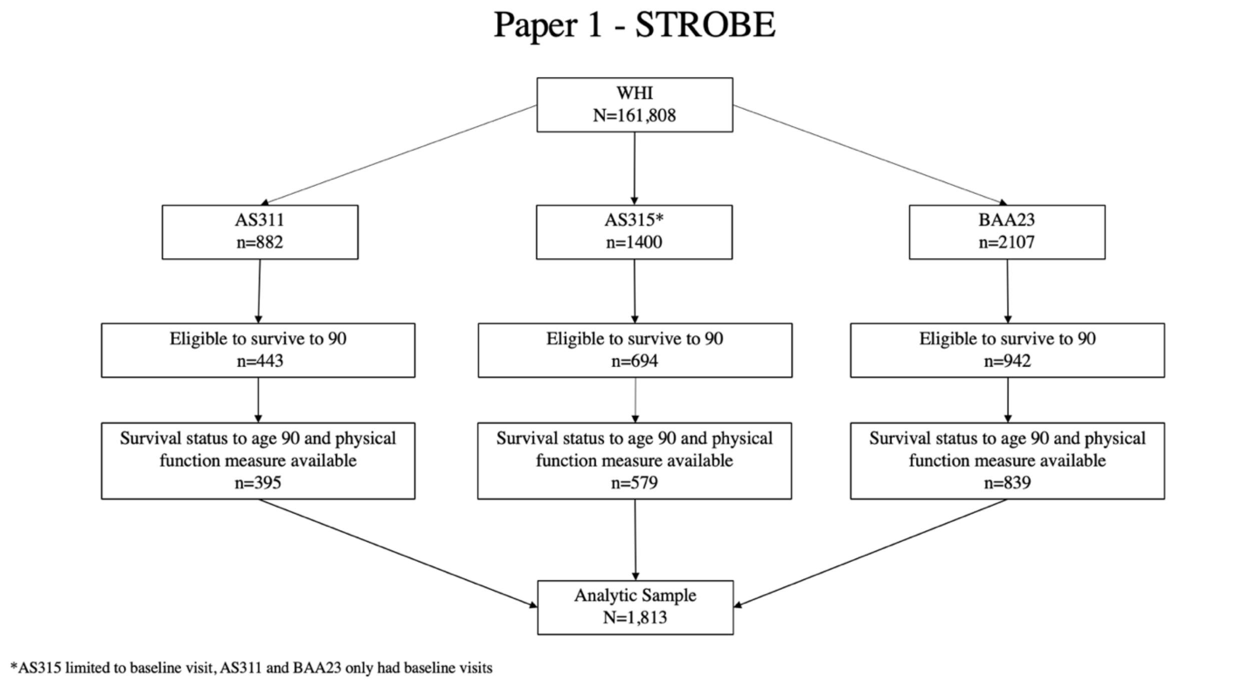

© 2022 Jain P et al. *JAMA Network Open.*

eFigure 2. Correlation of DNA Methylation Measures

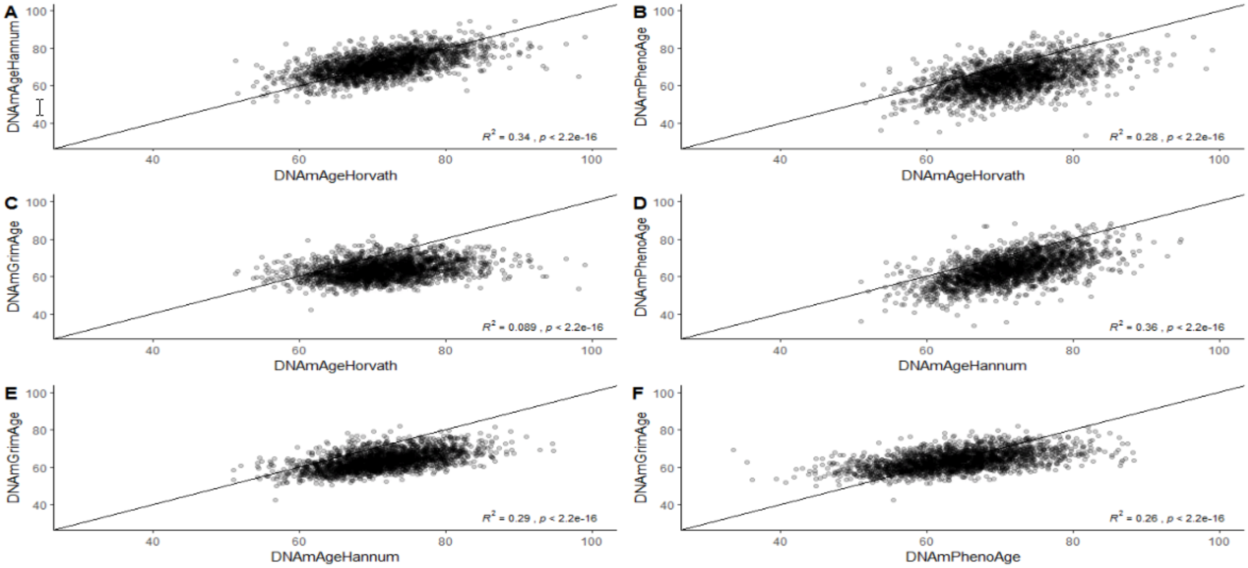

© 2022 Jain P et al. *JAMA Network Open.*

eFigure 3. Forest Plots of Association of Epigenetic Age Acceleration Measures and Healthy Longevity by Ancillary Study

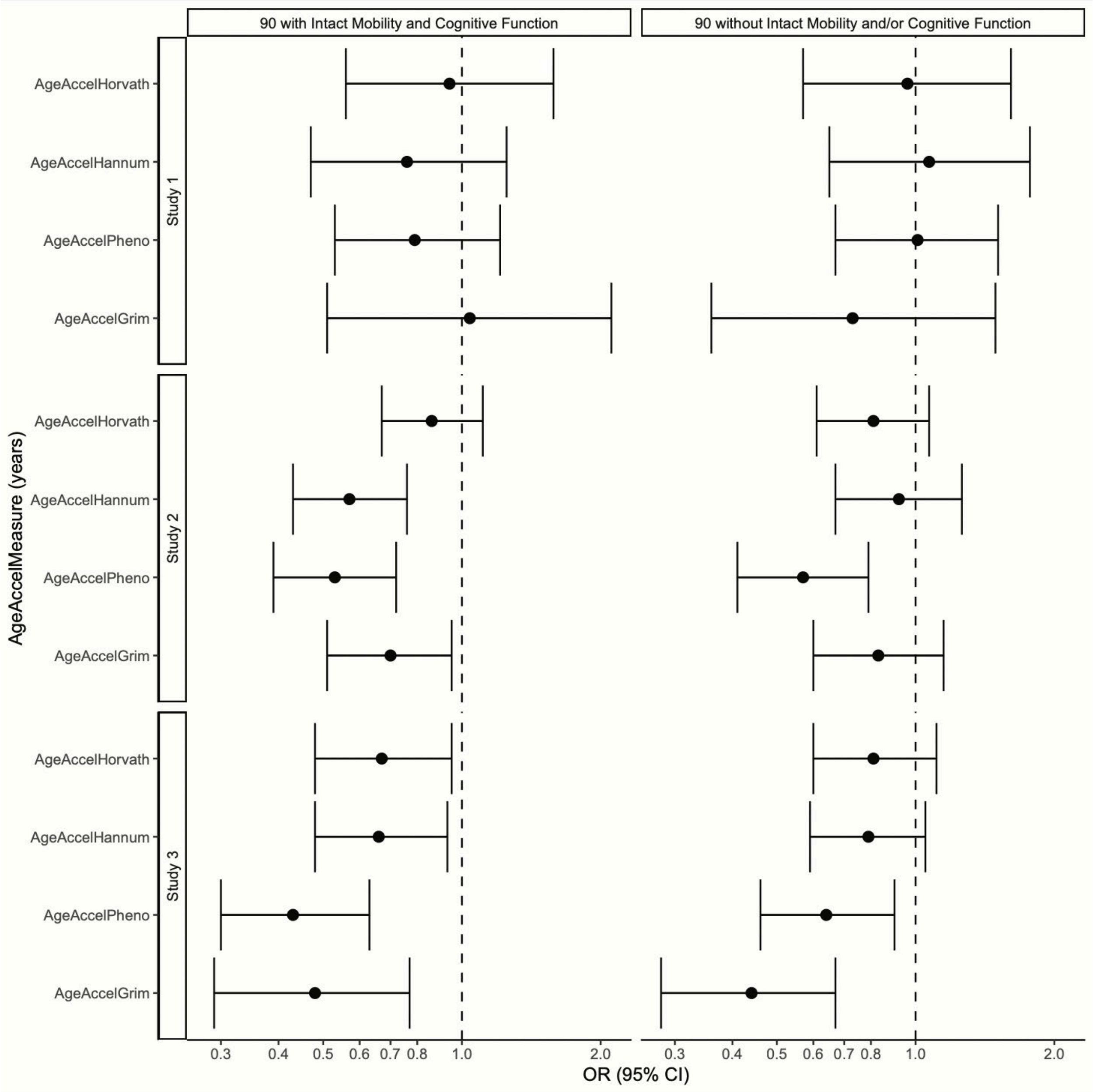

© 2022 Jain P et al. *JAMA Network Open.*
